# Increase in Chronic Medications and Polypharmacy—The Multifaceted Burden of COVID-19 Disease on Public Health Care

**DOI:** 10.3390/jpm13091321

**Published:** 2023-08-28

**Authors:** Antonella Gallo, Marcello Covino, Alice Lipari, Simona Pellegrino, Francesca Ibba, Maria Chiara Agnitelli, Matteo Tosato, Francesco Landi, Massimo Montalto, Gemelli against COVID-19 Post-Acute Care Team

**Affiliations:** 1 Department of Geriatrics and Orthopedics, Fondazione Policlinico Universitario “A. Gemelli”, IRCCS, Università Cattolica del Sacro Cuore, Largo A. Gemelli, 8, 00168 Rome, Italymatteo.tosato@policlinicogemelli.it (M.T.); francesco.landi@unicatt.it (F.L.); massimo.montalto@unicatt.it (M.M.); 2 Department of Emergency Medicine, Fondazione Policlinico Universitario “A. Gemelli”, IRCCS, Università Cattolica del Sacro Cuore, Largo A. Gemelli, 8, 00168 Rome, Italy; marcello.covino@policlinicogemelli.it

**Keywords:** Long-COVID, polypharmacy, burden, public health

## Abstract

The long-term impact of COVID-19 disease is becoming a major global concern. In this retrospective monocentric analysis, we included consecutive subjects admitted to our COVID-19 Post-Acute Care Service for a SARS-CoV-2 infection that occurred between three and twelve months before. A home medication list relative to the period before SARS-CoV-2 infection (baseline) was recorded and compared with that one relative to the time of outpatient visit (follow-up). Drugs were coded according to the Anatomical Therapeutic Chemical Classification (ATC) System. In a total of 2007 subjects, at follow-up, a significant increase with respect to baseline was reported in the total median number of chronic medications (two [0–4] vs. one [0–3]) and in specific ATC-group drugs involving the alimentary, blood, cardiovascular, genitourinary, muscle–skeletal, nervous and respiratory systems. In a multivariate analysis, COVID-19 disease severity and age > 65 years resulted in the best predictors for an increase in the number of medications, while anti-SARS-CoV-2 vaccination played a significant protective role. The long-term care of patients infected by COVID-19 may be more complex than reported so far. Multidisciplinary and integrated care pathways should be encouraged, mainly in older and frailer subjects and for patients experiencing a more severe disease. Vaccination may also represent a fundamental protection against long-term sequelae.

## 1. Introduction

The coronavirus disease 19 (COVID-19) has caused more than 6,000,000 deaths worldwide, with a significant economic and socio-cultural impact all around the world [1]. More recently, attention has been focused on the clinical and public health sequelae of the acute phase of SARS-CoV-2 infection. A multitude of long-lasting symptoms, such as breathlessness, cognitive impairment, abdominal discomfort, fatigue, anxiety and depression have been reported so far [2,3,4].

Recently the World Health Organization agreed to defining “Long-COVID” (or Post-COVID condition) as the continuation or development of new symptoms three months after the initial SARS-CoV-2 infection, with these symptoms lasting for at least two months with no other explanation [4].

The prevalence of Long-COVID has varied across and within many countries, ranging from 1 to over 75% [5,6]. Studies assessing hospitalized patients have typically reported higher prevalence estimates, even ranging from 32.6% to 87% [6]. Given the considerable number of individuals who experienced a COVID-19 disease worldwide, the Long-COVID condition has been recently defined as a second pandemic [7,8,9], leading to a medical and socioeconomic burden, and also to detrimental consequences on social life [7,8,9].

In recent years, there has been a growing interest in the topics of “polypharmacy” and “pharmacologic burden”, particularly for older and frailer patients [10,11,12,13,14,15]. A modification in drug regimen, also involving an increase in the total number of medications, is often reported after an acute phase of different conditions, mainly after discharge from the hospital [10,11,12]. Medical investigations during hospitalization may reveal previously unknown pathologies; other times, the disease itself can lead to an organ failure, which requires a more intense medication regimen for secondary preventions, such as for the cardiovascular system.

Although this evidence is strong enough in a context of discharge from hospitalization, conversely, the same data may be very difficult to obtain for diseases managed in an outpatient setting.

More recently, also based on continuously updating experiences with COVID-19 infection, new knowledge is emerging about the “post-acute infection syndromes (PAISs)” [16], i.e., the occurrence of chronic sequelae in a proportion of exposed individuals following some infections, mainly by viral agents. Despite the underlying mechanisms and etiologic factors being quite unknown or not having been sufficiently explained, there is consensus about their substantial health care and social impact in the long term. Nevertheless, as for COVID-19 disease, data on the specific pharmacological burden are very limited so far.

In a large previous retrospective work on patients who survived a COVID-19 infection in Lombardy during the first wave, during a three-month follow-up period, Mannucci et al. [17] reported a significant increase in the number of metabolic, hematological, cardiovascular, neuropsychiatric and respiratory drug classes when compared with a corresponding period of 2019, before the pandemic’s outbreak. In particular, polypharmacy was more frequent in patients hospitalized in medical wards and particularly in the intensive care unit (ICU).

However, starting from the wild-type strain infection, the SARS-CoV-2 virus has spread around the globe, leading to the identification of different viral variants, characterized by variable transmission patterns, virulence and severity of disease symptoms. Moreover, vaccine protection and the evidence that symptom frequency may diminish over time from the infection [18,19,20], but not completely disappear [21], require a detailed assessment to be carried out by a longer follow-up.

In our study, we investigated the potential long-term pharmacological burden in patients who recovered from the acute phase of COVID-19 infection throughout the different years of the pandemic.

## 2. Patients and Methods

This retrospective study was conducted in the context of “COVID-19 Post-Acute Care Service” at the Fondazione Policlinico Universitario “Agostino Gemelli” IRCCS of Rome, Italy. The access to this service was scheduled before discharge for those patients hospitalized for a COVID-19 infection in our hospital that wanted to check their condition for any consequence and was able to perform a follow-up visit (not bedridden, not a nursing home resident). Instead, for outpatients, the access was guaranteed by a voluntary request by mail. These requests could be sent directly by the patient, or by their General Practitioners. Patients were offered a comprehensive assessment including collection of a detailed medical history and a thorough physical examination. A multidisciplinary approach, encompassing internal medicine, geriatric, ophthalmological, otolaryngologist, pneumological, cardiological, neurological, immunological and rheumatological evaluations, was adopted to explore all possible consequences of SARS-CoV-2 infection. Demographic and clinical data, including medication assessment, characteristics of the COVID-19 disease and vaccination status, were collected in a structured electronic data-collection system.

### 2.1. Study Sample and Design

We searched the electronic database of our COVID-19 Post-Acute Care Service to identify consecutive subjects visiting our Service from October 2020 and February 2023 with a confirmed SARS-CoV-2 infection which occurred between three and twelve months before. All personal data were replaced by a univocal numerical code and the work was carried out on an anonymous database.

The list of drugs regularly taken at home in the period before SARS-CoV-2 infection (baseline) was recorded and compared with that one relative to time of visit to our Post-COVID Service (follow-up). Drugs were coded according to the Anatomical Therapeutic Chemical (ATC) Classification System [21]. Firstly, the total number of medications was defined as the number of used original drug compounds (fifth level ATC code: chemical substance) for both evaluations. Then, for the most prescribed classes in our population, belonging to the “A-Alimentary tract and metabolism”, “B-Blood and blood forming organs” and “C-Cardiovascular system” ATC index, we performed a more detailed analysis until the second level. Drugs not included in the ATC classification, as well as homeopathic products or dietary supplements, were not considered for the analysis.

The number of used drugs was classified as ‘no polypharmacy’ 0–4 drugs and ‘polypharmacy’ ≥ 5 drugs. Moreover, the need for chronic oxygen supplementation was recorded both for baseline and for the time of the follow-up visit.

The presence of main chronic diseases, including heart disease (heart failure, ischemic cardiomyopathy), hypertension, diabetes mellitus, chronic renal disease, pulmonary disease, active cancer and autoimmune diseases, was recorded for both baseline and follow-up evaluations.

Regarding SARS-CoV-2 infection, the following data were collected: date of infection, time from infection to our Service admission (days) and need for oxygen supplementation and/or intensive care unit (ICU) admission during the acute phase of COVID-19 disease. According to the year of COVID-19 infection (2020, 2021 and 2022), subjects were further divided into three subgroups. Anti-SARS-CoV-2 vaccination status was also recorded for each patient at time of follow-up visit.

We excluded all patients for which all these data were not retrievable or absent. We also excluded those subjects who were hospitalized for further acute illness occurring in the follow-up period.

This study was approved by the Università Cattolica and Fondazione Policlinico Gemelli IRCCS Institutional Ethics Committee (IRB#001300820). All patients attending our “COVID-19 Post-Acute Care Service” signed a consent at admission to use personal data for research purposes.

### 2.2. Statistical Analysis

Categorical variables were presented as numbers and percentages. Continuous data were presented as medians (inter-quartile range). Categorical variables were statistically compared using the Chi-square test for unpaired comparisons, and McNemar’s test for paired comparisons. Continuous variables were compared using the Mann–Whitney U test for two-group analyses, and Kruskal–Wallis ANOVA in cases of three or more groups. Paired analysis was made with the Wilcoxon signed-rank test.

For time-dependent analysis, the time was calculated in days from the COVID-19 diagnosis (baseline), and Cox regression analyses were used. Risk curves were estimated with the Kaplan–Meier methods, and differences in risk/survival were assessed using the log-rank test. Significant variables at univariate analysis were entered into a multivariate Cox regression model to identify independent risk factors for the evaluated endpoints. The results of the Cox regression analysis are reported as Hazard Ratio (HR) (95% confidence interval).

A two-sided *p*-value of 0.05 or less was considered significant in all the analyses. All data were analyzed with SPSS v26^®^ (IBM, New York, NY, USA).

## 3. Results

### 3.1. Sample Study

We reviewed 2200 records relative to patients attending our “COVID-19 Post-Acute Care Service” during the study period for a SARS-CoV-2 infection which occurred in the last three to twelve months. One-hundred and ninety-three incomplete records were excluded. Thus, our final study cohort included 2007 subjects (989 females, 1018 males) with a median age of 55 [46–64] years. Demographic and clinical data, information about COVID-19 disease severity and SARS-CoV-2 vaccination status at admission to the “COVID-19 Post-Acute Care Service” are reported in Table 1, both relative to the total cohort and to the different subgroups stratified by year of infection.

### 3.2. Changes in Drug Use and Rate of Main Chronic Disease Diagnosis before and after SARS-CoV-2 Infection

The total median number of daily drugs regularly taken by our study subjects before their SARS-CoV-2 infection was one [0–3], compared with a median number of two [0–4] drugs, taken daily three to twelve months later (*p* < 0.001). Also, the percentage of subjects on polypharmacy at the follow-up evaluation was significantly increased compared with baseline (*p* < 0.001).

A comparison between baseline and follow-up evaluation according to the different classes of drugs dispensed to our total cohort is reported in Table 2. In particular, a significant increase in medications compared with baseline was reported for drugs belonging to ATC classes A, B, C, G, H, M, N and R (*p* < 0.001). For the most frequently prescribed drugs in our population, included in ATC classes A, B and C, the further evaluation to the second level of the ATC classification is reported in Table 2.

For drugs in the A class, both for “Drugs for acid related disorders” (A02) and “Drugs used in diabetes” (A10), we found a significant increase (*p* < 0.001 for both the second level classes). The same results were found for drugs in the second level of the B class, which are “Antithrombotic agents” (B01), and for drugs belonging to the second level of the C class, represented by “Antihypertensives (C02), “Diuretics” (C03), “Beta-blocking agents” (C07), “Calcium channel blockers” (C08), “Agents acting on the renin-angiotensin system” (C09) and “Lipid modifying agents” (C10) (*p* < 0.001 for all the comparisons) (*p* < 0.001).

Compared with baseline, we found a significant increase at the time of visit to our COVID-19 Post-Acute Care Service in the rate of diagnosis of cardiac disease (5.9% vs. 6.8%), hypertension (28.5% vs. 32.7%), diabetes mellitus (8.6% vs. 9.8%) and pulmonary diseases (5.3% vs. 6.4%) (*p* < 0.001 for all the comparisons).

### 3.3. Factors Related to Increased Number of Drugs from Baseline

Overall, about one-third of subjects, 627 (31.2%), reported an increase in the number of daily drugs from baseline, while 1380 (68.7%) did not. The correlation of demographic data, clinical features and COVID-19 disease severity was evaluated via univariate and multivariate analysis (Table 3). In the univariate analysis, age (absolute value and over-65-years status), body mass index (BMI), time of infection, year of infection, vaccination, oxygen supplementation and ICU admission during the COVID-19 infection, were all correlated with an increased number of drugs from baseline (*p* < 0.05).

**Figure 1:**
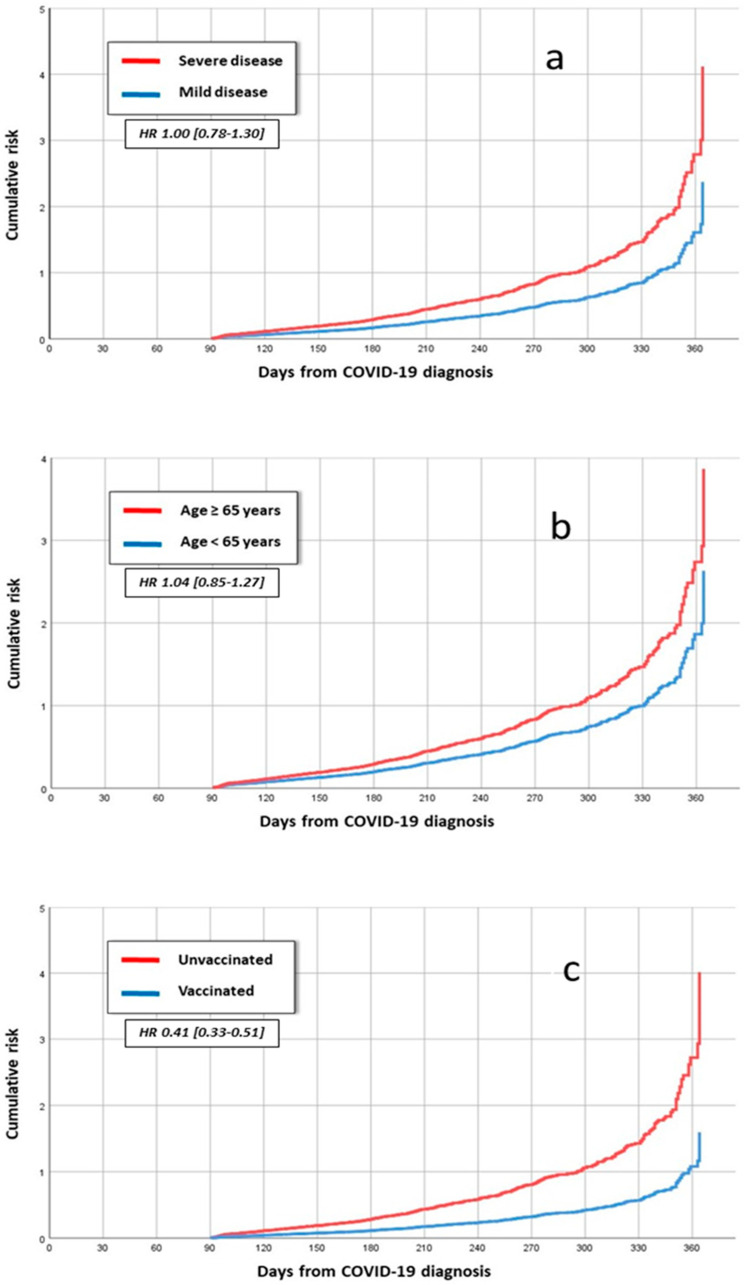
The multivariate-adjusted Cox regression analysis showed that COVID-19 disease severity (**a**) and age > 65 years (**b**) were independent risk factors for medication increase, while anti-SARS-CoV-2 vaccination was associated with a reduced risk of increase in drug prescription (**c**).

The multivariate-adjusted Cox regression analysis showed that COVID-19 disease severity and age > 65 years were independent risk factors for medication increase (Table 3, Figure 1a,b), while anti-SARS-CoV-2 vaccination was associated with a reduced risk of increase in drug prescription (Table 3, Figure 1c).

**Figure 2:**
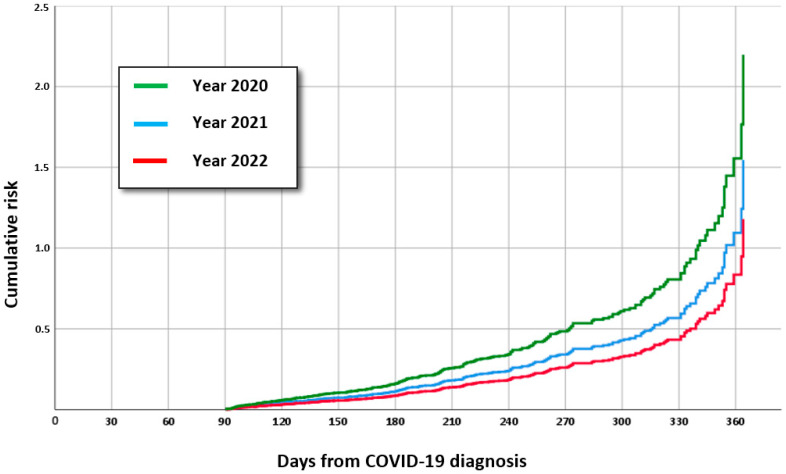
The multivariate-adjusted Cox regression analysis showed the most relevant increase in number of drugs from baseline was reported for those subjects who experienced a COVID-19 disease in 2020, while the least increase was reported for those subjects who experienced a COVID-19 disease in 2022.

Moreover, stratifying our sample size by year of infection, we found a significant difference among subjects infected during the three different years of the pandemic. In particular, the most relevant increase in the number of drugs from baseline was reported for those subjects who experienced a COVID-19 disease in 2020. Conversely, the least increase was shown for those subjects who experienced a COVID-19 disease in 2022 (Table 3, Figure 2).

## 4. Discussion

Although the exact numbers are unknown, it is believed that across the WHO European Region, more than 17 million people have developed symptoms that can be diagnosed as “Long COVID” during the first years of the pandemic [17,18,19,20,21]. Growing evidence shows that sequelae of SARS-CoV-2 infection may be responsible for another public health crisis [18,19,20,21]. Therefore, it is imperative to emphasize this situation and increase the awareness of physicians and patients.

A study by Mannucci et al. [17] reported that in individuals previously admitted to the ICU and medical wards during the first wave of infection, rehospitalizations, admission to hospital emergency rooms and outpatient medical visits were much more frequent in the 6-month period after SARS-CoV-2 negativization than in the same pre-pandemic period. The use of drugs and biochemical tests increased in all cases. Moreover, during the 3-month post-infection period, polypharmacy and daily use of medications belonging to the alimentary and metabolic (A), hematological (B), cardiovascular (C), nervous system (N) and respiratory (R) classes represented a common finding in these patients [17].

No similar data about specific changes in drug use are available throughout a longer follow-up after a COVID-19 infection.

Our cohort included patients attending a COVID-19 Post-Acute Care Service three to twelve months after SARS-CoV-2 infection, all of them able to perform an outpatient assessment, with a median age of 55 years. We also excluded those subjects who were hospitalized for further acute illness occurring in the follow-up period. Based on these conditions, it is likely that they may be representative of a relative well-being subgroup of the general population. However, even in this selected cohort, we confirm the results by Mannucci et al., showing that after SARS-CoV-2 infection, about one-third of subjects routinely took an increased number of drugs compared with the period before COVID-19 disease, even a year after disease occurrence. Also, in our study, the severity of COVID-19 disease was among the best predictors for polypharmacy and increased drug use.

The timing of assessment appears to be important, as different authors reported that symptom frequency can diminish over time from the infection [18,19]. However, the burden of symptomatic sequelae remained high and a remarkably lower health status than the general population was reported in COVID-19 survivors at 2 years [21,22,23]. We found that patients admitted to our Post-COVID Service six months and later since the date of infection showed a higher risk of increasing the number of drugs. It is possible that, as previously reported, while Long-COVID symptoms may almost disappear within a year, the organ dysfunctions occurring in the post-infectious period will have a significantly longer impact on general health status. Nonetheless, in our cohort, this result may be more conceivably explained by the fact that subjects undergoing a first visit six months and later since the infection had more likely experienced COVID-19 disease during the first years of the pandemic.

It is known that different variants may present with variable transmission patterns, virulence and severity of disease symptoms [21]. We compared subjects infected during the different years of the pandemic, showing that those ones who recovered from the acute phase of infection during 2020 were more likely at risk of increasing the number of daily drugs. A recent work by Mizrahi et al. showed that the wild-type, alpha and Delta variants resulted in similar long-term COVID-19 sequelae [24]. However, additional assessment of long-term outcomes of the Delta and the Omicron variants are still ongoing and only few data are available so far [25].

Our COVID-19 survivors came from the different stages of the global pandemic, so the results we found might reflect the long-term health outcomes of patients infected with different SARS-CoV-2 strains. We do not have data of the specific variants involved in our patients’ COVID-19 disease, so we cannot perform a detailed comparison among the different strains. However, it is conceivable that subjects experiencing the COVID-19 disease during 2020 were infected with the wild-type strain, unlike most of the subjects infected in 2021 and 2022.

Differences in our outcome through the subsequent years may undoubtedly be affected by vaccine development and global vaccination campaigns, which represent the cornerstone in protecting people from serious illness and preventing death [26,27,28]. We showed that vaccination status represents an independent variable, since unvaccinated subjects were more likely at risk of increasing the number of drugs after recovery of the acute phase of SARS-CoV-2 infection. This result may be firstly explained by the success of vaccination in drastically decreasing the risk of severe disease, also with new variants [29]. Also in our cohort, in fact, we found a significant decrease in the percentage of patients requiring oxygen supplementation or ICU admission over the three years of the pandemic.

To date, little data are available about the role of vaccination in the frequency and severity of Long-COVID symptoms and eventual differences among the wild-type and new variants [25,30]. Antonelli et al. showed that Omicron cases were less likely to experience Long-COVID for all vaccine timings with respect to Delta cases, also after stratification by age group [25].

Whether prior vaccination reduces cardiovascular, metabolic and respiratory COVID-19 sequelae is still under investigation. A retrospective cohort study by Al-Aly et al. showed that vaccinated participants (*n* = 16,035, mean age of 67 years, 91% male) were less likely to have at least one post-acute sequelae of COVID-19, including cardiovascular disorders, at 6 months compared with unvaccinated individuals (*n* = 48,536, mean age of 56 years, 86% male) [31]. In our smaller sample size, we can suppose a relationship among vaccination, a reduction in chronic post-acute sequelae and, consequentially, a reduction in the number of drugs. However, although encouraging, these results should be considered preliminary and may deserve confirmation in larger and longer multicentric studies.

The long-term impact of COVID-19 on cardiovascular health and mortality is becoming a major global concern. Acute cardiovascular complications of SARS-CoV-2 infection are well-described [32], and can lead to long-term sequelae requiring chronic cardiovascular care. A large analysis recently demonstrated an excess burden of cerebrovascular and cardiovascular disorders, such as new-onset hypertension and de novo heart failure, between 30 days and 12 months after COVID-19 infection in comparison to control cohorts, even in those who were not hospitalized for acute infection [32,33,34,35]. The mechanism for this predisposition towards atherosclerotic cardiovascular disease is not entirely clear, but is likely related to the proinflammatory effects of prothrombotic responses caused by viral infection [34,35].

Some authors also reported that COVID-19 survivors are at an increased risk of developing incident diabetes and consequent use of anti-hyperglycemic drugs [36]. Also, in our cohort we found a significant increase in the rate of cardiovascular and metabolic diagnosis during the follow-up period, with respect to baseline, as extrapolated by records of the multidisciplinary assessment. Therefore, we showed a significant increase in the use of cardiovascular and alimentary/metabolic drugs after SARS-CoV-2 infection. However, the design and results of our study cannot lead us to clearly support a direct relationship, which remains mainly speculative. In fact, if our findings may potentially, and partially, be explained by the direct and indirect cardiovascular and metabolic effects of the virus, it also has to be considered that patients with COVID-19 received more investigations and blood tests because of their illness, and this could lead to the formulation of new diagnoses, which would be otherwise delayed or unrecognized. Likewise, the lack of a detailed cardiologic or metabolic assessment before COVID-19 infection for most of our subjects did not give us the possibility to recognize a well-defined role of COVID-19 infection in the occurrence of eventual signs of organ dysfunctions during the follow-up.

The significant increase in proton pump inhibitor (PPI) usage that we found in our patients also deserves great relevance. Although we do not have data about eventual confirmed peptic ulcer disease for some of them, it is likely that a consistent percentage of our patients started PPI therapy after the acute infective episode without a clear clinical indication, as inappropriately happens in people who already receive other medications or in older patients discharged from internal medicine and geriatric wards [15]. This finding may be worthy of careful attention in long-term follow-up, not only due to the economic burden of PPIs, but in particular due to the health issues linked to an inappropriate prescription of PPIs and concern related to safety in long-term use [15].

Older age (>65 years) represented an independent risk factor for increasing the number of drugs during the follow-up. It has been reported that the number of used drugs may rise with age, tending to stagnate or decrease after approximately 90 years of age; however, this change has been estimated to be between 0.04 and 0.48 drugs annually [37]. Compared with these data, we found a median greater increase in all our cohort, being more significant in the older categories. These frailer and comorbid subgroups should probably need more global public efforts to adequately face the impact of both acute COVID-19 disease and the burden of its long-term sequelae.

Our work has some limitations. Firstly, this is a retrospective mono-centric study. Therefore, in spite of the large sample size, our results could not be broadly generalized and should be confirmed in larger and longer multicentric studies. Secondly, subjects admitted to a Post-COVID Service were more likely to complain of Long-COVID symptoms with respect to the general population, which may have overestimated the prevalence of Long-COVID syndrome. On the other hand, they also were patients able to perform an outpatient follow-up and we excluded those who were further re-hospitalized to avoid a bias in drug dispensation. However, it is unlikely that these selection criteria led to an underestimation of the prevalence of the acute severe form of SARS-CoV-2 infection, as revealed by the significant number of patients undergoing oxygen supplementation or admitted to the ICU during the acute phase of infection. Finally, for most of the enrolled subjects, the list of drugs and comorbidities, both relative to baseline and follow-up, was recorded based on self-reported details given by the patients or their care-givers at the first access to our Post-COVID Service. Therefore, similar to most follow-up studies of COVID-19, an information bias was possible, although our history-taking usually enclosed revision of electronic health records and previous visit reports, when available.

## 5. Conclusions

In conclusion, our work showed that the long-term care of patients recovered from a SARS-CoV-2 infection might be more complex than reported so far, with a significant burden on public health, not only as regarding long-lasting symptoms, but also for the eventual consequences of the multiorgan involvement of the disease. Multidisciplinary and integrated care pathways should be encouraged, mainly for patients experiencing a more severe disease, or in older and frailer subjects. Vaccination is confirmed as the best protection against severe disease, and may play a relevant role also against long-term sequelae.

## Figures and Tables

**Table 1:** Baseline demographic and clinical features and COVID-19 disease severity of total cohort and different subgroups according to year of infection.

	Total Cohort (n = 2007)	Year of Infection			*p*
		2020 (n = 1017)	2021 (n = 673)	2022 (n = 317)	
Demographic and clinical features					
Median age (years)	55 [46–64]	56 [47–65]	56 [46–64]	49 [42–59]	<0.001
Age > 65 years (N, %)	489 (32.2%)	269 (26.5%)	165 (24.4%)	55 (17.5%)	0.05
Sex (females, %)	989 (49.2%)	464 (45.6%)	324 (48%)	201 (49.3%)	<0.001
Median body mass index (kg/m^2^)	25.9 [23.1–29.1]	25.9 [23.4–28.9]	26.2 [29.3–29.4]	24.7 [21.6–28.7]	<0.001
Cardiac disease (N, %)	118 (5.9%)	64 (6.3%)	41 (6.1%)	13 (4.1%)	0.34
Hypertension (N, %)	571 (28.5%)	316 (31.1%)	190 (28.1%)	65 (20.6%)	0.002
Diabetes mellitus (N, %)	172 (8.6%)	96 (9,4%)	58 (8.6%)	16 (5.1%)	0.05
Pulmonary disease (N, %)	107 (5.3%)	67 (6.6%)	33 (4.9%)	7 (2.7%)	0.09
Chronic kidney disease (N, %)	56 (2.8%)	29 (2.9%)	24 (3.6%)	3 (1.0%)	0.07
Active cancer (N, %)	24 (1.2%)	13 (1.3%)	8 (1.2%)	3 (1.0%)	0.90
Autoimmune disease (N, %)	50 (2.5%)	13 (1.3%)	17 (2.5%)	23 (7.3%)	<0.001
COVID-19 disease					
Oxygen supplementation (N, %)	900 (44.8%)	490 (24.4%)	392 (19.5%)	18 (0.9%)	<0.001
ICU admission (N, %)	228 (11.3%)	128 (12.6%)	98 (14.5%)	2 (0.6%)	<0.001
Time of admission at Post-COVID Service					
Median time from infection (days)	170 [121–239]	143 [109–192]	199 [131–279]	206 [169–252]	<0.001
SARS-CoV-2 vaccination (yes, %)	606 (30.2%)	36 (3.5%)	294 (43.8%)	276 (87.6%)	<0.001

**Table 2:** Changes in drug use before and after SARS-CoV-2 infection.

Parameters	Total Cohort (N = 2007)		*p*
Median number of drugs	1 [0–3]	2 [0–4]	<0.001
Polypharmacy (N, %)	255 (12.1%)	375 (18.7%)	<0.001
Chronic oxygen supplementation	2 (0.1%)	45 (2.2%)	<0.001
Use of drug classes by ATC levels (N, %)			
A (Alimentary tract and metabolism) - Drugs for acid related disorders (A02) - Drugs used in diabetes (A10)	504 (25.1%)	712 (35.5%)	<0.001
	265 (13.2%)	392 (19.5%)	<0.001
	172 (8.6%)	212 (10.5%)	<0.001
B (Blood and blood forming organs) - Antithrombotic agents (B01)	284 (14.2%)	364 (18.2%)	<0.001
	270 (13.5%)	349 (17.4%)	<0.001
C (Cardiovascular System) - Antihypertensives (C02) - Diuretics (C03) - Beta-blocking agents (C07) - Calcium channel blockers (C08) - Agents acting on the renin-angiotensin system (C09) - Lipid modifying agents (C10)	721 (35.9%)	835 (41.6%)	<0.001
	36 (1.8%)	38 (1.9%)	<0.001
	190 (9.5%)	241 (12.0%)	<0.001
	276 (13.8%)	367 (18.3%)	<0.001
	239 (10.9%)	242 (12.1%)	<0.001
	466 (23.2%)	489 (24.4%)	<0.001
	276 (13.8%)	304 (15.1%)	<0.001
D (Dermatological)	7 (0.3%)	10 (0.5%)	0.37
G (Genitourinary system and sex hormones)	154 (7.3%)	189 (9.4%)	<0.001
H (Systemic hormonal preparations, excl. sex hormones and insulins)	272 (13.6%)	305 (15.2%)	<0.001
J (Anti-infective for systemic use)	9 (0.4%)	12 (0.6%)	0.51
L (Antineoplastic and immunomodulating agents)	32 (1.6%)	35 (1.7%)	0.26
M (Musculo-skeletal system)	77 (3.8%)	112 (5.6%)	<0.001
N (Nervous system)	196 (9.8%)	290 (14.5%)	<0.001
P (Antiparasitic products, insecticides and repellents)	3 (0.1%)	5 (0.2%)	0.16
R (Respiratory system)	84 (4.2%)	142 (7.1%)	<0.001
S (Sensory organs)	26 (1.3%)	32 (1.6%)	0.06
V (Various)	1 (0%)	3 (0.1%)	0.16

**Table 3:** Univariate and multivariate analysis about factors correlated to increased number of drugs after SARS-CoV-2 infection.

Variables	Increased Number of Drugs (N = 627)	Stable or Reduced Number of Drugs (N = 1380)	Univ *p* Value	Multivariate HR (CI 95%)
Median age (years)	53.5 [45–63]	57 [48–67]	<0.001	
Age > 65 years (N, %)	309 (15.4%)	180 (9%)	0.002	1.04 (0.85–1.27)
Sex (females/males)	694/686	295/332	0.18	0.93 (0.78–1.09)
Median body mass index (kg/m^2^)	25.7 [22.9–28.7]	26.1 [23.4–29.6]	0.01	
Median time from infection (days)	168 [123–236.75]	177 [118–245]	0.58	
Vaccination (N, %)	436 (31.6%)	170 (27.1%)	0.02	0.41 (0.33–0.51)
Year of infection (N, %):				
- 2020 - 2021 - 2022	682 (49.4%)	335 (53.4%)	0.01	1.39 (1.04–1.86)
	467 (33.8%)	206 (32.9%)		
	231 (16.7%)	86 (13.7%)		
Oxygen supplementation	542 (39.3%)	358 (57.1%)	<0.001	1.00 (0.78–1.30)
ICU admission	119 (8.6%)	109 (17.4%)	<0.001	0.68 (0.53–0.87)

ICU: intensive care unit; Univ: univariate; HR: Hazard Ratio; CI: confidence interval.

## Data Availability

Not applicable.
